# Effect of content framing in smoking prevention campaigns on recognition and attitudes: a virtual reality study

**DOI:** 10.1080/21642850.2024.2420810

**Published:** 2024-11-22

**Authors:** Solenne Bonneterre, Oulmann Zerhouni, Maréva Barré, Reinout Wiers, Marilisa Boffo

**Affiliations:** a Laboratoire Parisien de Psychologie Sociale, Département de Psychologie, Université Paris Nanterre, Nanterre, France; b Université Clermont Auvergne, Clermont-Ferrand, France; c Université Rouen Normandie, Rouen, France; d University of Amsterdam, Amsterdam, Netherlands; e Erasmus School of Social and Behavioral Sciences, Rotterdam, Netherlands

**Keywords:** Tobacco, smoking, attitudes, prevention posters, health promotion campaigns, immersive virtual reality, incidental exposure

## Abstract

**Objectives:**

Anti-tobacco campaigns often suffer from a lack of systematic evaluation and may not always have the intended impact on the target population. Our research adopted immersive virtual reality (iVR) to systematically evaluate preventive anti-tobacco messages in a controlled setting while mimicking a naturalistic and ecological environment. We investigated the effect of content framing of Anti-tobacco posters on attitudes and cravings toward tobacco, and poster recognition.

**Methods:**

Participants were undergraduate students (n = 121), mostly female (76%). They were immersed in a virtual environment incidentally exposing them to either negatively, positively, or neutrally framed preventive posters based on their experimental condition. Their gaze was eye-tracked during the entire procedure.

**Results:**

Results indicate that incidental exposure to preventive anti-tobacco posters while directly looking at negatively framed posters was associated with lower positive attitudes toward tobacco. Incidental exposure to posters did not impact their explicit recognition, except when exposed to negatively framed posters. No significant effect was found on craving.

**Conclusions:**

We advise health campaign designers to consistently use negatively framed preventive messages and recommend the use of iVR to evaluate campaigns before launching them.

Tobacco smoking is one of the world's leading and most preventable causes of death, according to the World Health Organization (about 8 million people a year, WHO, 2022) and is responsible for severe health issues (cancer, cardiovascular and respiratory illnesses, Bonaldi et al., 2019). As such, Smoking is a major health problem and the target of several preventive interventions by public health authorities. A variety of preventive interventions have been developed, including social media advertising, billboard posters and public health campaigns such as STOPTOBER in England and Mois Sans Tabac in France (Beck et al., 2020; Chan et al., 2020). These campaigns focus primarily on smoking cessation, but they also have an impact on smoking prevention by raising awareness and motivating smokers to quit. While STOPTOBER and Mois Sans Tabac are designed to encourage smoking cessation, research has shown that preventive messages can be effective in increasing smokers’ awareness of health risks and their desire to quit (Durkin et al., 2012). Studies such as Bala et al. (2017) and Wakefield et al. (2010) further demonstrate the effectiveness of anti-tobacco campaigns in both prevention and cessation efforts, as these campaigns can reach diverse populations through multiple channels. Although the primary focus of these campaigns is on cessation, their preventive impact should not be overlooked. However, evaluating their efficacy in an ecological context presents several methodological challenges, limiting the internal and external validity of results (Anker et al., 2016; Noar et al., 2009). The goal of this study is to systematically evaluate the efficacy of poster-based preventive campaigns by addressing whether exposure to anti-tobacco billboard posters impacts tobacco attitudes, cravings, and their recognition, by using immersive virtual reality (iVR) to recreate the ecological setting in which posters are usually seen.

## Evaluation of prevention campaigns: why field and lab approaches are limited

Prevention campaigns are difficult to evaluate because of the diversity in content and channels to broadcast them (Chan et al., 2020) and the lack of standardization of best practices to evaluate them (Anker et al., 2016; Noar et al., 2009). The use of theoretical background (Glanz, 2010) to design and evaluate prevention campaigns directly with the targeted population has been called for (Atkin & Rice, 2012). Some campaigns have been declared effective based on arbitrary standards rather than on objective assessments, as shown by a few meta-analyses and systematic reviews concluding on the low effectiveness of prevention campaigns to reach persuasive goals, notably because of the lack of rigor in their evaluation (Anker et al., 2016; Chan et al., 2020; Noar et al., 2009; Noar et al., 2018).

On a more pragmatic side, the impact of prevention billboard posters is difficult to evaluate in the field. First, it is difficult  – if not impossible, to estimate the frequency of exposure (Atkin & Rice, 2012) and if and how individuals process their contents. More precisely, it is difficult to establish whether individuals process the contents of prevention messages in the environment reflectively, i.e. in a controlled, goal-directed fashion, or more reflexively, i.e. in a more automatic, involuntary fashion (i.e. outside of voluntary, conscious, efficient, or controlled effort; Hommel & Wiers, 2017; Korteling et al., 2018). Second, it is difficult to assess recognition and attitudes following exposure to preventive posters as they are embedded in daily life and consequently rife with confounding factors affecting content processing. Exposure to preventive posters in a realistic context is saturated with information (e.g. crowds, advertising, signs, traffic, noise, ongoing tasks). Individuals may not always be attentive to their environment and may treat messages incidentally (Earp et al., 2011, 2013). The evaluation of prevention campaigns, particularly those aimed at reducing tobacco use, can be less effective when conducted in controlled laboratory settings, as these environments often fail to capture the complexities of real-world contexts that influence individual responses. Instead, naturalistic settings provide a more accurate reflection of how preventive messages work in everyday life, allowing researchers to observe real-world responses and behaviors. Therefore, a combined approach that includes both laboratory and field studies is essential for understanding the effectiveness of anti-tobacco strategies. Multiple studies have pointed to the importance of these exposure conditions as they might have negative side effects, such as enhanced likability of tobacco instead of decreasing positive attitudes (e.g. Earp et al., 2011, 2013; Moorman & van den Putte, 2008). Exposure conditions significantly influence attitudes towards tobacco products, often increasing their favorability rather than decreasing positive perceptions, particularly among adolescents. Research shows that exposure to pro-tobacco media and environments correlates with increased susceptibility to smoking among adolescents, highlighting the need for targeted interventions to mitigate these effects (Fulmer et al., 2015). Furthermore, comprehensive tobacco control policies, such as smoke-free ordinances, have been shown to reduce exposure to tobacco smoke and improve public health outcomes, especially for vulnerable populations such as children (Carpenter et al., 2011; Pérez-Ríos et al., 2024).

## Incidental exposure to preventive billboard posters

Incidental exposure refers to being exposed to a stimulus without directly or consciously processing it and has been specially investigated in the brand marketing field (Biscaia et al., 2014; Breuer & Rumpf, 2012; Zerhouni et al., 2016, 2019). For example, in sports events individuals’ attention is usually directed at the match, while brand sponsorship is displayed in the surroundings. Exposure to brands is then incidental and has been found to lead to higher brand recall the longer the sponsor is visible (Biscaia et al., 2014; Breuer & Rumpf, 2012) and to more positive attitudes toward brands (Zerhouni et al., 2016, 2019). Similarly, we can assume that when walking in a street, individuals’ attention is focused on their goals (e.g. going from A to B, running errands, meeting others) rather than directly toward billboard posters displayed in the area. As such, individuals are more likely to be exposed to preventive posters incidentally. When exposed incidentally to preventive messages, individuals are more likely to encode them without consciously or deeply processing them (Hommel & Wiers, 2017; Korteling et al., 2018). Anti-tobacco messages would then activate the concept of smoking in memory and its evaluation (i.e. attitudes) while missing out the full propositional message (i.e. ‘no smoking’; Earp et al., 2011, 2013), leading to the potential risk of counterproductive effects (i.e. increased activation of the concept of ‘smoking’ and motivation to smoke) as shown in studies on alcohol prevention (Krank et al., 2010).

## How does the framing of prevention poster campaigns modulate their perception?

Health promotion campaigns usually include content-framed posters, either positively (i.e. highlighting the benefits of reducing or quitting an unhealthy behavior) or negatively (i.e. focusing on the negative effects of adopting or continuing an unhealthy behavior). However, a meta-analysis indicated that framing does not impact attitudes (Gallagher & Updegraff, 2012). A few studies have attempted to test the effect of exposure to anti-tobacco depending on its framing (e.g. Moorman & van den Putte, 2008), but never in the case of incidental exposure. Furthermore, to date, very few studies investigated the role of framing in prevention message recognition, with mixed results (e.g. positively framed messages are better recalled than negative ones, Masumoto et al., 2020; no effect of framing on recall, Mollen et al., 2017). And none of them has investigated message recognition under incidental exposure. We aimed to address this gap by comparing the effect of incidental exposure to different message framing (positive, negative, or neutral) on attitudes, cravings, and recognition of anti–Anti-tobacco posters.

## Immersive virtual reality to evaluate prevention campaigns

Immersive virtual reality (iVR) is a human–computer interface that recreates virtual environments (VE) in which users are immersed and can interact through various sensory channels (usually sight, but also touch, sound, smell). iVR is a device usually composed of a head-mounted display (HMD) covering the eyes which, together with other motion tracking devices (e.g. hand-held controllers) enable users to interact directly with the VE and its characters. Due to the high level of immersion afforded by the technology, iVR allows individuals to act the closest to real-life behavior (Parsons et al., 2017) and behave naturally in the VE by generating the feeling of presence (i.e. the ability to project oneself mentally into the VE as we would in real-life, Cummings & Bailenson, 2015; Sanchez-Vives & Slater, 2005; Slater, 2009). As an example, it is common to see iVR users fearing for their life while being in a VE displaying a plank and crossing a cliff (Bailenson, 2018). The more present, the more the user feels like the VE is the real world.

iVR appears as an ideal technology to help adress the challenges of evaluating health promotion campaigns in an ecological setting, while enabling a high degree of experimental control. Given the role that presence and immersion may play in the experience with the VE and the effects of exposure to its contents (i.e. the more present the user feels in the VE, the more vivid the experience, and the more memorable and persuasive this can be; Ahn et al., 2019; Bailenson, 2018; Cummings & Bailenson, 2015; Parsons et al., 2017) we also evaluated their impact on memorization, craving and attitude. Moreover, recent data has shown that serious games (Lau et al., 2017) as well as VR apparatus can be used to be evaluate billboard-based campaigns (Bonneterre et al., 2024; Schmälzle et al., 2023) as well as in cognitive training (Matheis et al., 2007; Nègre et al., 2023).

## The present study

Our main goal was to evaluate the efficacy of poster-based Anti-tobacco campaign s in an ecological setting using iVR technology. We conducted a study in which we evaluated the effect of prevention poster framing on the same outcomes, comparing negatively (i.e. underlying risks and negative consequences of smoking), positively (i.e. underlying benefits of quitting smoking), and neutrally framed (i.e. purely informative) messages. Variables associated with the use of iVR (i.e. presence, immersion, and cybersickness) were explored as covariates. We posited that (H1) when exposed to positive or negative (i.e. valenced) anti-tobacco posters, attitudes toward tobacco would be impacted accordingly (e.g. positive posters exposure would lead to more positive attitudes) in comparison to neutral posters; (H2) participants exposed to valenced posters would retain them better than participants exposed to neutral prevention posters, and (H3) being exposed to Anti-tobacco posters would elicit cravings independent of their valence. Cravings were included as predictor of attitudes and presence, immersion, and cybersickness were explored as covariates. The study was pre-registered at: https://osf.io/s3w6t.

## Method

### Participants

An a priori power analysis (Faul et al., 2007) was conducted assuming a medium effect size (*f^2^* = .15) for a multiple linear regression (fixed model, R^2^ increase with three key predictors: condition, two eye-tracking measures and their interaction), which recommended a minimum *N* of 108 to ensure 90% power for our design. These participants were recruited via an online post on the University website. Participants were recruited through [Masked for review] online participant pool for psychology studies. Participants were screened for eligibility by assessing if they had any perceptive problems (e.g. vision trouble), were prone to cybersickness, or had any disorder for which the use of iVR technology is not recommended (e.g. epilepsy). The study was approved by the Ethics Review Board of 2023-01-08.

The final sample included 121 participants (n = 41 in the negatively framed posters condition, n = 40 in each of the other conditions), on average 20.1 years old (SD = 2.95), mainly women (76%). Participants were mostly non-smokers (70.2%), followed by regular smokers (17.4%), occasional smokers (7.4%), and former smokers (5%). Of smokers, the majority were non-dependent to tobacco (86.7%), followed by heavily dependent (6.7%), lightly dependent (3.3%), and moderately dependent (3.3%). Most participants declared having used a VR device in the past (55.8%), but only a few (4.5%) felt sick when using it. No baseline differences were observed among the experimental groups concerning age, gender, and FTND scores (all *p*’s > .05, all descriptive statistics are available in Table 1).

**Table 1.** Descriptive statistics.

Gender		Women	Men	Total
Total N (%)		91 (77.1)	27 (22.9)	118
Age	Mean (SD)	19.9 (3.1)	20.2 (2.2)	20.0 (2.9)
IPQ	Mean (SD)	0.3 (0.7)	0.2 (0.7)	0.3 (0.7)
Immersion	Mean (SD)	4.7 (1.0)	4.7 (1.3)	4.7 (1.1)
Cybersickness	Mean (SD)	1.2 (0.2)	1.2 (0.2)	1.2 (0.2)
Attitude score	Mean (SD)	2.1 (1.2)	2.0 (1.3)	2.1 (1.3)
TCQ	Mean (SD)	1.9 (1.0)	1.8 (1.1)	1.8 (1.0)
Exposure Duration	Mean (SD)	522061.1 (213950.1)	457586.3 (136013.7)	507308.4 (200155.2)
Directed Gaze	Mean (SD)	139.1 (185.4)	142.0 (165.2)	139.8 (180.3)

### Measures and materials

#### iVR device and virtual environment

**Figure 1.**
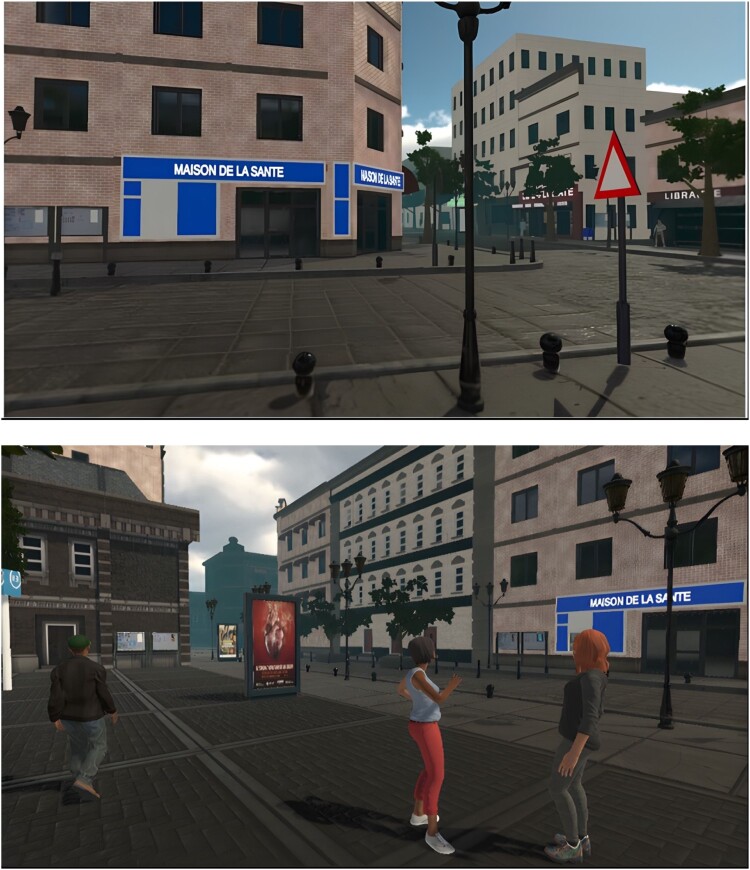
Screenshot of the VR environment.

The experiments took place in a Sensiks**®** VR pod, in which participants can sit and wear an Oculus Rift 2**®** and use two hand-held controllers to move around the VE. The VE depicted a Parisian suburb where billboards standing on the sidewalk or wall-mounted panels were displayed. Posters were randomly^1^ selected and displayed from a pool of billboards in the experimental condition with an equal amount of negative, positive, and neutrally framed posters. All billboards were blank in the control condition (see Figure 1 for screenshots).

#### Eligibility screening questionnaire

The eligibility screening included questions regarding participants’ physical ability to participate and three questions on smoking status: (1) did the participant use to be a smoker or is currently a smoker, and (2) if so, is s/he a regular or occasional smoker, and (3) for how long s/he smoked or used to smoke. For smoking participants, the FTND (Heatherton et al., 1991) was further used to assess severity of nicotine dependence. Response options range from 0 to 3 depending on the item, with a total score used to define four levels of dependence severity: non-dependent (0-2), low dependent (3-4), mildly dependent (5-6), highly dependent (≥7).

#### Presence

Presence was assessed using the French version of the iGroup Presence Questionnaire (IPQ; Viaud-Delmon, n.d.), a short, reliable, and widely used questionnaire (Schwind et al., 2019). The IPQ contains 14 questions rated on a scale from – 3 (completely disagree) to +3 (completely agree), such as ‘To what extent did the virtual world seem real to you?’.

#### Immersion

Immersion was assessed by questioning participants about VR device features (Cummings & Bailenson, 2015), complementing aspects not mentioned by the IPQ: ‘I was able to move into the virtual environment without thinking about how to use hand-held controllers’ and ‘I needed to or should have adjusted the helmet during the procedure’ on a 1 (completely disagree) to 7 (completely agree) Likert scale. Immersion was assessed as a manipulation check, aiming at evaluating the validity of our iVR set-up.

#### Attitudes toward tobacco

Participants indicated on a 7-point differential semantic scale their attitudes based on the following adjective pairs: risky/safe, not enjoyable/enjoyable, dislike/like, and bad/good (Stark et al., 2008). Ratings were averaged across all pairs, with the higher the score, the more favorable the attitudes toward tobacco.

#### Cravings for tobacco

The French version of the 12-item Tobacco Cravings Questionnaire (TCQ, Berlin et al., 2010) was used. Items (e.g. ‘I would do almost anything for a cigarette now’) were rated on a Likert scale from 1 (Strongly disagree) to 7 (Strongly agree).

#### Explicit recollection of prevention messages

A list of 30 billboard posters^2^ was presented to participants, asking whether they saw them during their walk in the VE. This list included 10 posters randomly selected from those presented in the VE and 20 distractors (i.e. posters that were not displayed in the VE). Recognition was correct when the participant indicated viewing posters that were presented in the VE and incorrect when indicating viewing posters that were not presented, or the other way around. Scores for correct recognition ranged from 1 to 10 and for incorrect recognition from 1 to 30. A total score was computed through the difference between correct minus incorrect recognition subs-cores, with the higher the score, the more individuals correctly recognized the posters (hence, a better recognition).

#### Eye-tracking measures

In the VE, participants’ gaze was constantly monitored with the eye-tracking technology in the HMD. Two scores were derived: Exposure Duration, indicating the total amount of time (in milliseconds) posters were in the field of view of the participant, independently of whether their gaze was directed at them (i.e. posters could also be in the peripheral view); and Directed Gaze, indicating to what extent participants directed their gaze toward posters (i.e. more sustained attention). The latter was computed automatically by the eye-tracking system for each participant and poster. Directed Gaze was progressively computed and added up every 0.2 s by calculating the percentage of the screen area a billboard took up and transforming it to a score from 0 to 20, which was added up to the current score (e.g. if a billboard occupied 10% of the screen, it was transformed to a score of 2, which was added up to the current total Directed Gaze). Consequently, the more participants had a poster in their field of view, the more it took up visual space and the higher the Directed Gaze score. For each participant, an average was made across all posters seen in the VE. For Exposure Duration, scores for each poster were instead summed up.

*Cybersickness*. Cybersickness was assessed using the 9-items Virtual Reality Sickness Questionnaire (VRSQ, Kim et al., 2018) to ensure the VE would not cause any cybersickness and discomfort. The scale assesses to what extent participants felt symptoms such as general discomfort, blurred vision, dizziness, etc. when using the iVR on a 4-point scale (none, slight, moderate, or severe).

### Procedure

Participants were recruited through the [Masked for review] online experiment platform. Participants first had to fill out the eligibility screening questionnaire. The sign-up required participants to complete an informed consent procedure where the experiment was described as a study aiming at studying space perception and movement within a virtual environment. This cover story was used to limit demand bias from the participants (see Corneille & Lush, 2022), ensuring they were exposed incidentally to anti-tobacco posters during their walk in the VE. At the end of the study, participants were debriefed and informed that the study was in fact about the exposure, perception, and recognition of anti–Anti-tobacco posters.

Eligible participants were randomly assigned to one of the conditions and exposed to 10 posters accordingly (i.e. positively, negatively, or neutrally framed). Billboard locations were randomized within participants. Participants started with a short tutorial in the iVR pod on how to use the device and the hand-held controllers to move into the iVR environment. Participants were then immersed in the VE. The experimenter verified first that the participant used the controller properly and guided them through the VE. Four short instructions were given for a duration of around 5 min of exposure: walking to the bus stop, reaching the end of the main street, seeking a red-headed person (i.e. virtual agent), and walking the street from the right to left. Participants were encouraged to move around at their own pace. Upon fulfilling the instructions, participants could spend some more time in the VE if they wanted to. After the iVR exposure, they completed the post-intervention measures, starting with the presence, immersion, and cybersickness questionnaires, followed by tobacco attitudes, craving and the message recognition measures. with the addition of one instruction in the VE (i.e. going all the way up the main street, then coming back to the starting point in the middle of the VE) to allow them to fully complete the VE.

### Data analysis

We conducted three hierarchical linear regression models, one for each outcome (i.e. attitudes, cravings, and recognition scores), with condition and the two eye-tracking scores, and their interactions, as main predictors. Age, gender, presence, immersion, cybersickness, and FTND scores were entered as baseline covariates in all models. We conducted a one-sample t-test for the recognition score in the experimental condition to ensure participants did not complete the task randomly. Finally, Exposure Duration scores were centered using a Z-score^3^ to reduce multicollinearity, which was not necessary for Directed Gaze as it was already a transformed score (whereas Exposure Duration was a sum of raw data). Two simple contrasts for Condition were included in all models to compare the valenced conditions to the neutral one and examine their interaction with the eye-tracking measures.

## Results

### Manipulation checks on VR use

Our procedure only slightly elicited presence (M = 0.31, SD = 0.72) and did not elicit cybersickness (M = 1.23, SD = 0.22). The degree of immersion was around average (M = 4.74, SD = 1.09), but was lower concerning the ease to use the controllers (M = 2.86, SD = 1.64). No group difference emerged on any of these variables (all *p*’s > .05).

### Hypothesis 1: The effect of valenced posters on tobacco attitudes

After controlling for covariates, the inclusion of the key predictors explained an additional 5% (*p* = .08) and 16% (*p* = .01) of the attitude and recognition score variance, respectively (see Tables 2 and 3). No main effect of condition appeared for tobacco attitudes.

**Table 2.** Hierarchical linear regression models for tobacco attitudes, controlling for participants’ demographic and VR covariates.

Predictor variables	*β*	*CI 95%*	*t*	*p*
Presence	−0.03	[−0.25, 0.19]	−0.30	.77
Immersion	−0.06	[−0.20, 0.09]	−0.77	.44
Cybersickness	0.20	[−0.52, 0.91]	0.54	.59
Cravings	0.97	[0.79, 1.15]	10.53	<.001
Age	0.03	[−0.02, 0.08]	1.06	.29
Gender	−0.04	[−0.40, 0.33]	−0.20	.84
FTND	−0.03	[−0.20, 0.13]	−0.39	.70
*Model 1*: R² = 0.60, AIC = 293, BIC = 293, *F*(7, 107) = 23.2, *p *< .001				
Presence	−0.04	[−0.26, 0.18]	−0.39	.70
Immersion	−0.01	[−0.16, 0.14]	−0.18	.86
Cybersickness	0.50	[−0.24, 1.24]	1.34	.18
Cravings	0.99	[0.81, 1.17]	10.77	<.001
Age	0.05	[0.00, 0.11]	1.94	.06
Gender	0.02	[−0.35, 0.39]	0.11	.91
FTND	−0.07	[−0.24, 0.10]	−0.79	.43
Condition: Negative – Neutral	−0.40	[−0.93, 0.13]	−1.51	.13
Condition: Positive – Neutral	−0.02	[−0.52, 0.47]	−0.10	.92
Exposure Duration	0.12	[−0.23, 0.46]	0.67	.50
Directed Gaze	−0.02	[−0.04, 0.00]	−2.32	.02
Condition (Negative-Neutral)*Exposure Duration	−0.45	[−0.93, 0.02]	−1.90	.06
Condition (Positive-Neutral)*Exposure Duration	0.07	[−0.35, 0.48]	0.32	.75
Condition (Negative-Neutral)*Directed Gaze	0.02	[−0.01, 0.04]	1.18	.24
Condition (Positive-Neutral)*Directed Gaze	0.02	[−0.01, 0.04]	1.35	.18
*Model 2*: R² = 0.65, AIC = 294, BIC = 340, *F*(15,99) = 12.5, *p *< .001 ΔR² = 0.05, *F*(8,99) = 1.83, *p *= .08				

Note. Exposure Duration = total time in milliseconds the posters were in the field of view; Directed Gaze = averaged running sum of the percentage of area billboards took up of the screen; FTND = Fagerstrom Test for Nicotine Dependence.

**Table 3.** Hierarchical linear regression models for poster recognition, controlling for participants’ demographic and VR covariates.

Predictor variables	*β*	*CI 95%*	*t*	*p*
Presence	−0.39	[−1.25, 0.46]	−0.91	.36
Immersion	0.32	[−0.24, 0.89]	1.13	.26
Cybersickness	−0.73	[−3.52, 2.06]	−0.52	.61
Age	0.13	[−0.07, 0.34]	1.28	.20
Gender	1.94	[0.51, 3.38]	2.69	.008
FTND	0.02	[−0.51, 0.55]	0.07	.94
*Model 1*: R² = 0.10, AIC = 605, BIC = 627, *F*(6,108) = 2.01, *p *= .07				
Presence	−0.54	[−1.38, 0.30]	−1.28	.20
Immersion	0.16	[−0.40, 0.72]	0.56	.57
Cybersickness	−2.32	[−5.13, 0.49]	−1.64	.11
Age	0.06	[−0.14, 0.27]	0.63	.53
Gender	1.80	[0.39, 3.21]	2.54	.01
FTND	0.11	[−0.44, 0.66]	0.40	.69
Condition: Negative-Neutral	2.09	[0.08, 4.10]	2.07	.04
Condition: Positive-Neutral	−1.05	[−2.92, 0.82]	−1.12	.27
Exposure Duration	−0.18	[−1.48, 1.11]	−0.28	.78
Directed Gaze	0.06	[−0.10, 0.14]	1.75	.08
Condition (Negative-Neutral)*Exposure Duration	0.91	[−0.89, 2.70]	1.01	.32
Condition (Positive-Neutral)*Exposure Duration	0.21	[−1.35, 1.76]	0.26	.79
Condition (Negative-Neutral)*Directed Gaze	−0.08	[−0.19, 0.02]	−1.55	.12
Condition (Positive-Neutral)*Directed Gaze	0.01	[−0.07, 0.10]	0.26	.80
*Model 2*: R² = 0.26, AIC = 599, BIC = 643, *F*(14,100) = 2.45, *p *= .005 ΔR² = 0.16, *F*(8,100) = 2.61, *p *= .01				

Note. Exposure Duration = total time in milliseconds the posters were in the field of view; Directed Gaze = averaged running sum of the percentage of area billboards took up of the screen; FTND = Fagerstrom Test for Nicotine Dependence.

### Hypothesis 2: Poster framing and recognition

**Figure 2.**
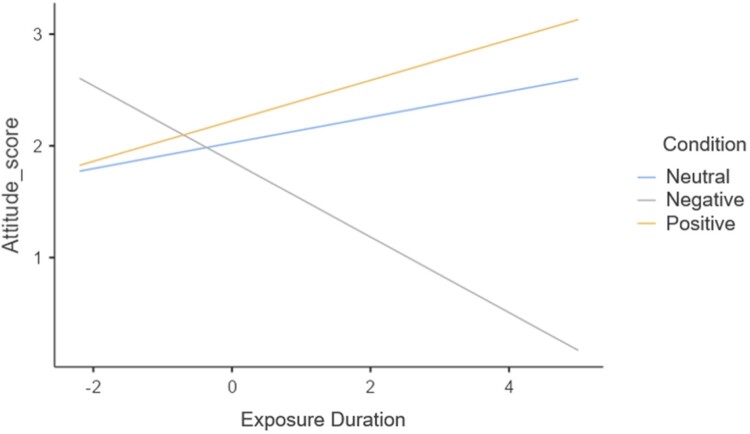
Interaction effect of Condition x Exposure Duration on tobacco attitudes scores.

Directed Gaze was found to negatively predict attitudes (*β *= −0.02 *p *= .02), i.e. the more individuals directly looked at the posters, the more negative the attitudes toward tobacco. The interaction effect between the negative vs neutral condition contrast and Exposure Duration (*β *= −0.45 *p *= .06) indicated that a longer presence of negatively framed posters in the field of view seemed to negatively impact attitudes to a larger extent compared to neutrally framed posters, albeit this effect was not significant (see Figure 2).

### Hypothesis 3: Exposure to posters and tobacco cravings

Regarding poster recognition, when posters were negatively framed participants memorized them better than purely informative ones (*β *= 2.07, *p *= .04). Also, women showed better recognition scores than men (*β *= 1.80, *p *= .01). No relevant effects were identified for craving (Table 4).

**Table 4.** Hierarchical linear regression models for poster craving, controlling for participants’ demographic and VR covariates.

Predictor variables	*β*	*CI 95%*	*t*	*p*
Presence	0.05	[−0.18, 0.28]	0.42	.68
Immersion	0.04	[−0.12, 0.19]	0.47	.64
Cybersickness	0.10	[−0.65, 0.85]	0.28	.78
Age	0.001	[−0.05, 0.06]	0.04	.97
Gender	−0.13	[−0.51, 0.26]	−0.66	.51
FTND	0.49	[0.35, 0.64]	6.85	< .001
*Model 1*: R² = 0.32, AIC = 303, BIC = 325, *F*(6,108) = 8.30, *p *< .001				
Presence	0.02	[−0.22, 0.26]	0.14	.89
Immersion	0.01	[−0.15, 0.17]	0.15	.88
Cybersickness	0.03	[−0.78, 0.83]	0.06	.95
Age	<−0.01	[−0.06, 0.06]	−0.01	.99
Gender	−0.14	[−0.54, 0.26]	−0.69	.50
FTND	0.49	[0.33, 0.65]	6.21	< .001
Condition: Negative-Neutral	0.11	[−0.46, 0.69]	0.39	.69
Condition: Positive-Neutral	−0.41	[−0.94, 0.13]	−1.52	.13
Exposure Duration	0.06	[−0.31, 0.43]	0.30	.77
Directed Gaze	−0.01	[−0.03, 0.01]	−0.79	.43
Condition (Negative-Neutral)*Exposure Duration	−0.02	[−0.54, 0.49]	−0.09	.93
Condition (Positive-Neutral)*Exposure Duration	−0.17	[−0.62, 0.28]	−0.76	.45
Condition (Negative-Neutral)*Directed Gaze	<0.01	[−0.03, 0.03]	0.26	.80
Condition (Positive-Neutral)*Directed Gaze	0.02	[−0.00, 0.04]	1.58	.12
*Model 2*: R² = 0.35, AIC = 312, BIC = 356, *F*(14,100) = 3.92, (*p *< .001) ΔR² = 0.03, *F*(8,100) = 0.75, *p *= .65				

## Discussion

Our research aimed to evaluate the effects of differently framed Anti-tobacco posters on attitudes and cravings for tobacco, as well as recognition of the posters in an environmental setting using immersive virtual reality (iVR). Specifically, we examined the effects of positive, negative, or neutral framing on these outcomes.

We found that poster content framing alone was not a predictor of attitudes toward tobacco, in line with Gallagher and Updegraff (2012)’s findings where exposure to positive or negative preventive content did not impact attitudes. Yet, while no direct univariate effect on attitudes was observed when poster framing was systematically manipulated, paying attention to posters seems to be associated with more negative attitudes toward tobacco, at the net of all relevant covariates. Yet, the size of the effect is very small (beta < 0.10).

Results further indicated that the longer negative posters were in participants’ field of view, the less positive their attitudes were compared to exposure to neutral posters. This suggests that using negatively framed posters and ensuring that individuals view them for a sufficient amount of time may be an effective strategy for influencing attitudes by reinforcing the negative consequences of smoking. For example, Bafunno et al. (2020) and Noar (2006) highlight the importance of placing anti-tobacco messages in channels that are widely viewed by the target audience to maximize exposure and effectiveness. Similarly, WHO's tobacco control guidelines recommend that high-quality anti-tobacco campaigns should last more than three weeks to ensure long-term engagement and message retention (WHO, 2020). The WHO Framework Convention on Tobacco Control (FCTC) further emphasizes the need for high-frequency, long-term campaigns to achieve optimal results (WHO FCTC, 2020).

In terms of poster recognition, our results challenge previous assumptions by showing that consistent exposure to negatively framed posters resulted in better recognition compared to neutrally framed posters. This finding underscores the potential efficacy of consistent negative messages in the recognition of prevention content.

Presence, immersion, and cybersickness were assessed to evaluate the effects of the iVR setup. Our protocol elicited moderate levels of presence and immersion, which did not affect any outcomes. However, the study revealed that higher cybersickness was associated with lower recognition scores, highlighting the importance of considering cybersickness in iVR research due to its potential impact on environmental perception and cognitive processing.

Despite certain limitations, such as the lack of a ‘pure’ control condition and the need for replication, our study provides valuable insights into the design of billboard-based prevention campaigns and the role of message framing in memory recognition and attitude change.

### Limitations

This study presents some limitations, the first one being that we only assessed explicit attitudes and recognition, although some studies recommend measuring both implicit and explicit (Herrmann et al., 2011). In the same fashion, we did not assess smoking behavior nor intentions to smoke, quit or reduce smoking. Even though these measures are used as outcomes in health prevention studies (e.g. Anker et al.,^ 2016^), they were not relevant for our research as our protocol deployed a too short exposure to the VE to have an impact on behavior or intentions. In fact, we believe that repeated exposure over a longer time can significantly impact such variables (Atkin & Rice, 2012; Glanz, 2010). Therefore, our studies only allowed us to estimate momentary effects of exposure to smoking prevention posters, and sustained, medium  – and longer-term effects of repeated exposure should also be explored.

Billboard posters were sourced from existing Anti-tobacco campaign s in our urban environment (i.e. billboards used in previous Anti-tobacco campaign s). Therefore, the experimental conditions were not as rigorously equivalent in terms of poster content compared to if we had created all posters ourselves for the study. Nonetheless, the use of existing prevention posters supports the ecological validity of our design as content used in the real world was used and presented under virtually similar conditions. Similarly, billboards across conditions only displayed anti–Anti-tobacco messages and no other unrelated content (e.g. ads, events, etc.) as usually found in real life. Although our virtual environment achieved a good level of realism, which represents a significant advance in the evaluation of health promotion campaigns, further improvements can be made to enhance its realism and applicability, more work is needed to bring realism to a yet higher level, improving both internal and external validity of our findings.

A relevant issue is the sample composed exclusively of students, relatively young, and mostly female non-smokers, whereas smokers were for the most part very lightly dependent on nicotine. It would be interesting to include more participants dependent on smoking as nicotine dependency may lead to a difference in message processing (Moorman & van den Putte, 2008). We may also have more insights into the role of cravings (Gass et al., 2014), as the present study is non-informative about the effect of prevention on smoking cravings.

Another limitation of our study is that we did not assess craving prior to iVR exposure, nor did we include a control group without poster exposure. This makes it difficult to conclude whether exposure to the posters directly induced craving. In addition, given that the majority of our participants were non-smokers, it is possible that the hypothesized effect of poster exposure on craving was not fully applicable to our sample. Future research should include pre  – and post-exposure measures of craving, as well as a control group, to better assess the potential influence of anti-tobacco posters on craving.

## Conclusions

This paper presented the first study using iVR to systematically evaluate preventive anti-tobacco campaigns in an ecological setting. It gave some evidence that directly looking at negative posters may lead to more negative attitudes toward tobacco. Hence, consistently using negatively framed messages seems a better strategy to ensure a negative impact on attitudes toward tobacco. Further, incidental exposure seems too short to leave a strong impression in explicit memory, except for consistently negatively framed posters. Replication is needed to corroborate these findings and the evaluation of prevention campaigns would need to focus on billboard size and location to see if and how these impact attention to the posters, and in turn their recognition.

Finally, our study shows how iVR can be a useful and ideal tool to systematically evaluate the design of population-based Health promotion campaigns. iVR offers a highly ecologic and realistic setting to evaluate how individuals process and may be impacted by health-related content embedded in their daily environment in a more standardized fashion, enabling the design of more impactful and efficacious health promotion campaigns. To more thoroughly examine the effects of anti–Anti-tobacco posters on craving, future studies should include both a baseline measure of craving and a control group that is not exposed to smoking-related content. This would allow for a clearer understanding of whether poster exposure, particularly among smokers, significantly influences craving levels and whether nonsmokers are also affected by smoking-related cues in a virtual environment.

## Data Availability

The data that support the findings of this study are openly available in at: https://osf.io/s3w6t.

## Notes

1 In all three conditions, 15 billboard locations were available. In the experimental condition, 10 of the 15 available billboard locations were randomly picked to display prevention posters. Among the list of 30 possible posters, 10 were randomly picked to be displayed randomly across the 10 selected billboards. The 5 remaining billboards were filled with the blank picture shown in the control condition. The randomization of posters and Billboard locations in which posters were displayed was done within participants.

2 Posters were based on existing Anti-tobacco posters. An original pool of 45 posters was presented to 388 participants in a previous online survey aimed at evaluating perceived persuasiveness and relevance of the posters. Additionally, participants had to indicate whether the posters were considered preventive (i.e., negatively framed), informative (i.e., neutrally framed) or promoting tobacco cessation (i.e., positively framed). Descriptive analyses were conducted to rank and select the final list of 30 posters (10 for each of the mentioned categories, see Appendix A for details).

3 We used a Z-score on Exposure Duration to reduce multicollinearity, which was not necessary for Directed Gaze as it was already a transformed score (whereas Exposure Duration was a sum of raw data).

## Appendix A: Posters used in the VR environments

The 30 posters used in the study varied in size, ranging from approximately 380 × 542 pixels to 600 × 800 pixels, ensuring good visibility in the virtual environment. The color schemes differed according to the framing: negative posters used dark and alarming colors such as black, red, and gray to emphasize the dangers of smoking; neutral posters used more balanced tones such as gray and light blue; and positive posters used bright and uplifting colors such as green, blue, and yellow to encourage smoking cessation. In terms of content, negative posters combined graphic images (e.g. diseased lungs) with minimal but powerful text such as ‘Smoking Kills,’ neutral posters were more text-heavy and offered factual information about the risks of smoking, and positive posters used uplifting images (e.g. healthy people) paired with motivational phrases such as ‘Quit Smoking Today’. This mix of visuals and text was intended to engage participants emotionally and cognitively, and we will include this detailed description in the manuscript to clarify the design and implications of the stimuli used.

## References

[CIT0001] Ahn, S. J., Hahm, J. M., & Johnsen, K. (2019). Feeling the weight of calories: Using haptic feedback as virtual exemplars to promote risk perception among young females on unhealthy snack choices. *Media Psychology*, *22*(4), 626–652. 10.1080/15213269.2018.1492939 32863775 PMC7453386

[CIT1001] Anker, A. E., Feeley, T. H., McCracken, B., & Lagoe, C. A. (2016). Measuring the effectiveness of mass-mediated health campaigns through meta-analysis. *Journal of Health Communication*, *21*(4), 439–456. 10.1080/10810730.2015.1095820 26953782

[CIT0002] Atkin, C. K., & Rice, R. E. (2012). Advances in public communication campaigns. In A. N. Valdivia (Ed.), *The international encyclopedia of media studies*. Blackwell Publishing Ltd. 10.1002/9781444361506.wbiems129.

[CIT1002] Bafunno, D., Catino, A., Lamorgese, V., Del Bene, G., Longo, V., Montrone, M., Pesola, F., Pizzutilo, P., Cassiano, S., Mastrandrea, A., Ricci, D., Petrillo, P., Varesano, N., Zacheo, A., & Galetta, D. (2020). Impact of tobacco control interventions on smoking initiation, cessation, and prevalence: A systematic review. *Journal of Thoracic Disease*, *12*(7), 3844–3856. 10.21037/jtd.2020.02.23 32802466 PMC7399441

[CIT0003] Bailenson, J. (2018). *Experience on demand: What virtual reality is, how it works, and what it can do*. WW Norton & Company.

[CIT0004] Bala, M. M., Strzeszynski, L., & Topor-Madry, R. (2017). Mass media interventions for smoking cessation in adults. *Cochrane Database of Systematic Reviews*, *11*, CD004704. 10.1002/14651858.CD004704.pub4 29159862 PMC6486126

[CIT0005] Beck, F., Guignard, R., & Richard, J. B. (2020). Mois sans tabac: évaluation d’une campagne de santé publique sur le tabagisme en France. *Revue des Maladies Respiratoires*, *37*(5), 421–429.

[CIT0006] Berlin, I., Singleton, E. G., & Heishman, S. J. (2010). Validity of the 12-item French version of the tobacco craving questionnaire in treatment-seeking smokers. *Nicotine & Tobacco Research*, *12*(5), 500–507. 10.1093/ntr/ntq039 20335281 PMC2902858

[CIT0007] Biscaia, R., Correia, A., Ross, S., & Rosado, A. (2014). Sponsorship effectiveness in professional sport: An examination of recall and recognition among football fans. *International Journal of Sports Marketing and Sponsorship*, *16*(1), 2–18. 10.1108/IJSMS-16-01-2014-B002

[CIT0008] Bonaldi, C., Boussac, M., & Nguyen-Thanh, V. (2019). Estimation du nombre de décès attribuables au tabagisme, en France de 2000 à 2015. *Bull épidémiol hebd*, *15*, 278–284.

[CIT0009] Bonneterre, S., Zerhouni, O., & Boffo, M. (2024). The influence of billboard-based tobacco prevention posters on memorization, attitudes, and craving: Immersive virtual reality study. *Journal of Medical Internet Research*, *26*, e49344. 10.2196/49344 38980707 PMC11285084

[CIT0010] Breuer, C., & Rumpf, C. (2012). The viewer’s reception and processing of sponsorship information in sport telecasts. *Journal of Sport Management*, *26*(6), 521–531. 10.1123/jsm.26.6.521

[CIT0011] Carpenter, C. S., Postolek, S., & Warman, C. (2011). Public-place smoking laws and exposure to environmental tobacco smoke (ets). *American Economic Journal: Economic Policy*, *3*(3), 35–61. 10.1257/pol.3.3.35

[CIT0012] Chan, L., O’Hara, B., Phongsavan, P., Bauman, A., & Freeman, B. (2020). Review of evaluation metrics used in digital and traditional tobacco control campaigns. *Journal of Medical Internet Research*, *22*(8), e17432. 10.2196/17432 32348272 PMC7448186

[CIT0013] Corneille, O., & Lush, P. (2022). Sixty years after orne’s American psychologist article : A conceptual framework for subjective experiences elicited by demand characteristics. *Personality and Social Psychology Review*, 10.1177/1088868322110436 35801624

[CIT0014] Cummings, J. J., & Bailenson, J. N. (2015). How immersive is enough? A meta-analysis of the effect of immersive technology on user presence. *Media Psychology*, *19*(2), 272–309. 10.1080/15213269.2015.1015740

[CIT0015] Durkin, S., Brennan, E., & Wakefield, M. (2012). Mass media campaigns to promote smoking cessation among adults : An integrative review. *Tobacco Control*, *21*(2), 127–138. 10.1136/tobaccocontrol-2011-050345 22345235

[CIT0016] Earp, B. D., Dill, B., Harris, J., Ackerman, J., & Bargh, J. A. (2011). Incidental exposure to no-smoking signs primes craving for cigarettes: An ironic effect of unconscious semantic processing. *Yale Review of Undergraduate Research in Psychology*, *2*, 12–23. 10.1037/e525772013-002

[CIT0017] Earp, B. D., Dill, B., Harris, J. L., Ackerman, J. M., & Bargh, J. A. (2013). No sign of quitting : incidental exposure to “no smoking” signs ironically boosts cigarette-approach tendencies in smokers. *Journal of Applied Social Psychology*, *43*(10), 2158–2162. 10.1111/jasp.12202

[CIT0018] Faul, F., Erdfelder, E., Lang, A. G., & Buchner, A. (2007). G* power 3: A flexible statistical power analysis program for the social, behavioral, and biomedical sciences. *Behavior Research Methods*, *39*(2), 175–191. 10.3758/BF03193146 17695343

[CIT0019] Fulmer, E., Neilands, T. B., Dube, S. R., Kuiper, N. M., Arrazola, R. A., & Glantz, S. A. (2015). Protobacco media exposure and youth susceptibility to smoking cigarettes, cigarette experimentation, and current tobacco use among us youth. *PLoS One*, *10*(8), e0134734. 10.1371/journal.pone.0134734 26308217 PMC4550466

[CIT0020] Gallagher, K. M., & Updegraff, J. A. (2012). Health message framing effects on attitudes, intentions, and behavior: A meta-analytic review. *Annals of Behavioral Medicine*, *43*(1), 101–116. 10.1007/s12160-011-9308-7 21993844

[CIT0021] Gass, J., Motschman, C., & Tiffany, S. (2014). The relationship between craving and tobacco Use behavior in laboratory studies : A meta-analysis. *Psychology of Addictive Behaviors : Journal of the Society of Psychologists in Addictive Behaviors*, *28*), 10.1037/a0036879 25134054

[CIT0022] Glanz, B. (2010). The role of behavioral science theory in development and implementation of public health interventions. *Annual Review of Public Health*, *31*, 399–418. 10.1146/annurev.publhealth.012809.103604 20070207

[CIT0023] Heatherton, T. F., Kozlowski, L. T., Frecker, R. C., & Fagerstrom, K.-O. (1991). The fagerström test for nicotine dependence : A revision of the fagerstrom tolerance questionnaire. *British Journal of Addiction*, *86*(9), 1119–1127. 10.1111/j.1360-0443.1991.tb01879.x 1932883

[CIT0024] Herrmann, J.-L., Walliser, B., & Kacha, M. (2011). Consumer consideration of sponsor brands they do not remember : taking a wider look at the memorisation effects of sponsorship. *International Journal of Advertising*, *30*(2), 259–281. 10.2501/IJA-30-2-259-281

[CIT0025] Hommel, B., & Wiers, R. W. (2017). Towards a unitary approach to human action control. *Trends in Cognitive Sciences*, *21*(12), 940–949. 10.1016/j.tics.2017.09.009 29150000

[CIT0026] Kim, H. K., Park, J., Choi, Y., & Choe, M. (2018). Virtual reality sickness questionnaire (VRSQ): motion sickness measurement index in a virtual reality environment. *Applied Ergonomics*, *69*, 66–73. 10.1016/j.apergo.2017.12.016 29477332

[CIT0027] Korteling, J. E., Brouwer, A.-M., & Toet, A. (2018). A neural network framework for cognitive bias. *Frontiers in Psychology*, *9*), https://www.frontiersin.org/articles/10.3389fpsyg.2018.01561. 10.3389/fpsyg.2018.01561 PMC6129743 30233451

[CIT0028] Krank, M. D., Ames, S. L., Grenard, J. L., Schoenfeld, T., & Stacy, A. W. (2010). Paradoxical effects of alcohol information on alcohol outcome expectancies. *Alcoholism: Clinical and Experimental Research*, *34*, 1193–1200. 10.1111/j.1530-0277.2010.01196.x 20477773 PMC4164266

[CIT1003] Lau, H. M., Smit, J. H., Fleming, T. M., & Riper, H. (2017). Serious games for mental health: Are they accessible, feasible, and effective? A systematic review and meta-analysis. *Frontiers in Psychiatry*, *7*, 209. 10.3389/fpsyt.2016.00209 28149281 PMC5241302

[CIT0029] Masumoto, K., Shiozaki, M., & Taishi, N. (2020). The impact of age on goal-framing for health messages: The mediating effect of interest in health and emotion regulation. *PLoS One*, *15*(9), e0238989. 10.1371/journal.pone.0238989 32941521 PMC7498008

[CIT0030] Matheis, R. J., Schultheis, M. T., Tiersky, L. A., DeLuca, J., Millis, S. R., & Rizzo, A. (2007). Is learning and memory different in a virtual environment? *The Clinical Neuropsychologist*, *21*(1), 146–161. 10.1080/13854040601100668 17366282

[CIT1005] Mollen, S., Engelen, S., Kessels, L. T. E., & van den Putte, B. (2017). Short and sweet: The persuasive effects of message framing and temporal context in antismoking warning labels. *Journal of Health Communication*, *22*(1), 20–28. 10.1080/10810730.2016.1247484 27997285

[CIT0032] Moorman, M., & van den Putte, B. (2008). The influence of message framing, intention to quit smoking, and nicotine dependence on the persuasiveness of smoking cessation messages. *Addictive Behaviors*, *33*(10), 1267–1275. 10.1016/j.addbeh.2008.05.010 18584971

[CIT0033] Nègre, F., Lemercier-Dugarin, M., Kahn-Lewin, C., Gomet, R., Zerdazi, E.-H. M., Zerhouni, O., & Romo, L. (2023). Virtual reality efficiency as exposure therapy for alcohol use: A systematic literature review. *Drug and Alcohol Dependence*, *253*, 111027. 10.1016/j.drugalcdep.2023.111027 38006671

[CIT1006] Noar, S. M. (2006). A 10-year retrospective of research in health mass media campaigns: where do we go from here? *Journal of Health Communication*, *11*(1), 21–42. 10.1080/10810730500461059 16546917

[CIT0034] Noar, S. M., Bell, T., Kelley, D., Barker, J., & Yzer, M. (2018). Perceived message effectiveness measures in tobacco education campaigns : A systematic review. *Communication Methods and Measures*, *12*(4), 295–313. 10.1080/19312458.2018.1483017 31428217 PMC6699787

[CIT0035] Noar, S. M., Palmgreen, P., & Zimmerman, R. S. (2009). Reflections on evaluating health communication campaigns. *Communication Methods and Measures*, *3*(1-2), 105–114. 10.1080/19312450902809730

[CIT0036] Parsons, T., Gaggioli, A., & Riva, G. (2017). Virtual reality for research in social neuroscience. *Brain Sciences*, *7*(12), 42. 10.3390/brainsci7040042 28420150 PMC5406699

[CIT0037] Pérez-Ríos, M., Ahluwalia, J., Guerra-Tort, C., García, G., Rey-Brandariz, J., Mourino-Castro, N., Teijeiro, A., Casal-Fernández, R., Galán, I., Varela-Lema, L., & Ruano-Ravina, A. (2024). Towards stronger tobacco control policies to curb the smoking epidemic in Spain. *Clinical and Translational Oncology*, *26*(7), 1561–1569. 10.1007/s12094-024-03385-9 38347375 PMC11178643

[CIT0038] Pianzola, F., Riva, G., Mantovani, F., & Kukkonen, K. (2020). Presence, flow, and narrative absorption : An interdisciplinary theoretical exploration with a new spatiotemporal integrated model based on predictive processing. *PsyArXiv*. o. 10.12688/openreseurope.13193.2 PMC10446082 37645177

[CIT0039] Sanchez-Vives, M. V., & Slater, M. (2005). From presence to consciousness through virtual reality. *Nature Reviews Neuroscience*, *6*(4), 332–339. 10.1038/nrn1651 15803164

[CIT0040] Schmälzle, R., Lim, S., Cho, H. J., Wu, J., & Bente, G. (2023). Examining the exposure-reception-retention link in realistic communication environments via VR and eye-tracking: The VR billboard paradigm. *PLoS One*, *18*(11), e0291924. 10.1371/journal.pone.0291924 38033032 PMC10688884

[CIT0041] Schwind, V., Knierim, P., Haas, N., & Henze, N. (2019). Using presence questionnaires in virtual reality. *Proceedings of the 2019 CHI Conference on Human Factors in Computing Systems*, 1–12. 10.1145/3290605.3300590

[CIT0042] Slater, M. (2009). Place illusion and plausibility can lead to realistic behaviour in immersive virtual environments. *Philosophical Transactions of the Royal Society B: Biological Sciences*, *364*(1535), 3549–3557. 10.1098/rstb.2009.0138 PMC2781884 19884149

[CIT0043] Stark, E., Borgida, E., Kim, A., & Pickens, B. (2008). Understanding public attitudes toward tobacco harm reduction: The role of attitude structure 1. *Journal of Applied Social Psychology*, *38*(10), 2615–2635. 10.1111/j.1559-1816.2008.00406.x

[CIT0045] Viaud-Delmon, I. (n.d.). iGroup presence questionnaire. igroup.org. http://igroup.org/pq/ipq/download.php

[CIT0046] Wakefield, M. A., Loken, B., & Hornik, R. C. (2010). Use of mass media campaigns to change health behaviour. *Lancet*, *376*(9748), 1261–1271. 10.1016/S0140-6736(10)60809-4 20933263 PMC4248563

[CIT1008] World Health Organization (WHO). (2020). Anti-tobacco mass media campaigns. https://www.who.int/data/gho/data/indicators/indicator-details/GHO/gho-tobacco-control-anti-tobacco-mass-media-campaigns

[CIT0047] World Health Organization. (2022, May 24th). Tobacco. World Health Organization (WHO). https://www.who.int/news-room/fact-sheets/detail/tobacco

[CIT1009] World Health Organization Framework Convention on Tobacco Control. (2020). Global progress in implementation of the WHO FCTC: Report by the Convention Secretariat. https://fctc.who.int/news-and-resources/publications/i/item/fctc-cop9-5

[CIT0050] Zerhouni, O., Bègue, L., Duke, A. A., & Flaudias, V. (2016). Dynamic exposure to alcohol advertising in a sports context influences implicit attitudes. *Alcoholism: Clinical and Experimental Research*, *40*(2), 422–428. 10.1111/acer.12966 26842261

[CIT0051] Zerhouni, O., Bègue, L., & O’Brien, K. (2019). How alcohol advertising and sponsorship works : effects through indirect measures. *Drug and Alcohol Review*, 10.1111/dar.12929 31037783

